# Human-Animal Medicine: Clinical Approaches to Zoonoses, Toxicants and Other Shared Health Risks

**DOI:** 10.3201/eid1606.100367

**Published:** 2010-06

**Authors:** Corrie Brown

**Affiliations:** University of Georgia, Athens, Georgia, USA

**Keywords:** Zoonoses, toxicants, human–animal medicine, veterinary medicine, book review

Hooray, finally a book emerges about the human–animal interface that addresses both perspectives equitably and seamlessly. Peter Rabinowitz, a physician, and Lisa Conti, a veterinarian, effectively present material that is thorough, balanced, and of great relevance for practitioners of all varieties of medicine.

More than half the pages comprise reports on each of 35 zoonoses. At the beginning of each report are key points divided into professional categories—public health professionals, human health clinicians, and veterinary clinicians—ensuring relevance for multiple readers. In addition, 55 pages deal with toxicoses, including environmental, gaseous, poisonous plants, herbicides/pesticides, and envenomations. Clinical signs, symptoms, species comparisons, treatment and prevention for these toxicoses are all spelled out clearly.

This inclusive approach, with its plentiful and accurate technical information, might be enough to justify purchasing the book for the shelf of any human or veterinary medical practice, but it is the additional 175 pages that set this volume apart from all others on the subject. A lengthy introductory chapter discusses the general concept of one medicine and why that concept does not mean one practitioner but rather integration of practitioners from multiple sectors. The chapter describes the serious legal and ethical considerations associated with professionally crossing the human–animal interface. The occupational health of animal workers is covered in detail and includes not only zoonotic agents that immediately come to mind, but also allergens, use of live vaccines, noise, anesthetic gases, and the psychosocial impacts of such issues as euthanasia. Animal-assisted therapy is covered in detail. The book includes a particularly useful section on immunocompromised persons and their exposure to animals and another one on animal bites. There is even a segment on travel, including concerns about wild animal contact, as well as disease hazards for pets that travel. These additional chapters make parts of this book relevant for a much wider audience that could potentially include policy makers, regulators, students, and academicians.

Throughout, the book is graphically pleasing, with text broken regularly by subheadings, tables, pen-and-ink drawings, algorithms, photographs, and intriguing side bars. Some of the side bars are case studies, with interesting scenarios and quick tips for promoting health.

